# The presurgical T staging of non-small cell lung cancer: efficacy comparison of 64-MDCT and 3.0 T MRI

**DOI:** 10.1186/s40644-015-0050-4

**Published:** 2015-09-03

**Authors:** Wei Tang, Ning Wu, Han OuYang, Yao Huang, Li Liu, Meng Li

**Affiliations:** Department of Diagnostic Radiology, Cancer Hospital, Chinese Academy of Medical Sciences, Peking Union Medical College, Beijing, China

**Keywords:** Lung cancer, Computed tomography, X ray, Magnetic resonance

## Abstract

**Background:**

Lung cancer has been the main concern of the cancer-related deaths worldwide. Non-small cell lung cancer (NSCLC) is reported the most common subtype of lung cancer. Initial staging of NSCLC is highly associated with the choice of treatment and prognosis of the patients. This study aims to prospectively compare the diagnostic efficacies of 64-multidetector-row computed tomography (MDCT) and 3.0 T magnetic resonance imaging (MRI) in T staging of NSCLC.

**Methods:**

Institutional review board approval and informed consent were obtained. Forty-five patients diagnosed with NSCLC who underwent preoperative MRI and MDCT scans were enrolled in the study. The efficacies of determination of T staging on MRI and MDCT were compared by using the McNemar test.

**Results:**

Of 45 patients diagnosed with NSCLC, the primary tumors were correctly staged in 38 (84.4 %) patients on MDCT, and in 37 (82.2 %) patients on MRI. There was no statistically significant difference between the two modalities in the overall T staging of NSCLC with the reference of pathological findings (*p* = 0.564). However, MDCT was indicated more accurate in determination of NSCLC staged T1 and T2 (100 % vs 75 %, 96.4 % vs 82.1 %), whereas MRI was presented slightly superior in identification of NSCLC staged T3 and T4 (80 % vs 50 %, 100 % vs 33.3 %).

**Conclusion:**

Both MDCT and MRI provided acceptable overall accuracies in determination of T staging in NSCLC. Furthermore, MRI was presented slight superiority for the advanced-stage tumors (i.e., NSCLC staged T3 and T4), whereas MDCT was indicated mild acceptance for the limited-stage tumors (i.e., NSCLC staged T1 and T2).

## Background

Lung cancer is one of the most common malignancies in China, with the cancer-related deaths reported rapidly over thirty years [1]. In 2008, lung cancer accounted for 14.5 % of all newly diagnosed malignancies in the USA [2], 85% of diagnosed lung cancers were non-small cell lung cancer (NSCLC) [3]. Surgical resection remains the curable approach among the treatments of choice in the management of NSCLC, despite the continuous improvement and development in the theraputic treatments so far. Initial TNM staging is important in the decision-making process of management as well as in predicting prognosis of the patients with NSCLC. [4].

Various imaging modalities have been used for preoperative T staging of NSCLC. Computed tomography (CT) has been considered as standard of reference for preoperative evaluation of intrathoracic spread of lung cancer [5]. However, the assessment of preoperative T staging in lung cancer on CT is still not totally satisfying, owing to its limitations in evaluation of tumor invasion into adjacent structures [6]. On the other hand, magnetic resonance imaging (MRI) has been increasingly utilized in preoperative staging of lung cancerdue to its strength in excellent soft tissue contrast and high spatial resolution [5]. The purpose of our study was to compare the efficacies of 3.0-T MRI and 64-MDCT for determining T staging of NSCLC.

## Methods

### Patients

The institutional review board approved this prospective study, and written informed consent were obtained from all patients. From July 2011 to February 2013, a total of 51 consecutive patients pathologically diagnosed with NSCLC who underwent MRI and MDCT examinations were enrolled in this study. Of these 51 patients, 6 were excluded owing to the history of preoperative neoadjuvant chemotherapy (n = 4) and intolerable claustrophobia occurred during the scanning (n = 2). The remaining 45 patients (30 men and 15 women; age range, 32-70 years; mean age, 56 years) were included in this study. MDCT and MRI scans were performed within a 7-day interval for each patient. Each case enrolled was imaged with an extent from the thoracic inlet through the adrenal glands either on MDCT or MRI.

### Image acquisition

MR examinations were performed on a 3.0-T MR scanner (SIGNA EXCITE HD, GE Medical Systems) with a dedicated 8-channel coil array system. All the patients were performed with serial sequences: axial T_1_-weighted fast spin echo (FSE) (TR = 600-900 ms, TE = 5.8 ms, acquisition matrix size = 288 × 192 pixels), axial, sagittal and coronal breath-hold T_2_-weighted FSE (TR = 6000-8000 ms, TE = 85 ms, acquisition matrix size = 288 × 244 pixels), axial breath-hold T_2_-weighted FSE with fat suppression (TR = 6000-8000 ms, TE = 85 ms, acquisition matrix size = 288 × 244 pixels). Other scanning parameters included were as follows: field of view = 36-38 cm, slice thickness = 7-8 mm with a 1 mm interslice gap. Dynamic contrast-enhanced MR images were obtained with a breath-hold T1-weighted in-phase two-dimensional gradient-echo sequence (TR = 2.9 ms, TE = 1.3 ms, TI = 5.0 ms, field of view = 40-42 cm, slice thickness = 4 mm with 0 mm interslice gap, acquisition matrix size = 288 × 192 pixels,flip angle = 15°). A bolus of 20 ml gadolinium diethylene triaminepenta acetate (Gd-DTPA) as the intravenous contrast agent was administered for all patients at a rate of 3 ml/sec. Dynamic contrast-enhanced MR scan was started 5 s after the completion of the administration. Each scanning of three continuous phases was obtained with breath-hold in 15 s, and in between there was a 15 s internal. Totally 24 continuous phases of images were achieved in 4 min.

A64-MDCT scanner (GE LightSpeed VCT, GE Medical System) was used in this study. The scanning parameters were as follows: tube voltage of 120 kV, tube currents of 270 mA, detector configuration of 0.625 (detector collimation) x 64 (detectors), reconstruction section thickness of 1.25 mm, reconstruction section interval of 0.8 mm, rotation time of 0.6 s, and pitch of 0.984. A bolus of iohexol used as intravenous contrast agent was administered with intravenous infusion at a dose of 1.5 ml/kg body weight (average, 90-120 ml) at a rate of 3 ml/s. Contrast-enhanced CT images were obtained 35 s after completion of the administration.

### Imaging evaluation and reference standard

Images acquired on each modality including the multiplannar reconstruction images of MDCT (i.e., coronal and sagittal images) were evaluated. Two groups of 3 experienced radiologists (N.W., Y.H. and W.T., with 8-30 years of experience in thoracic CT imaging; H.OY., L.L. and M.L., with 5-20 years of experience in thoracic MRI) interpreted the MDCT manifestations and MR findings, respectively. Consensus was achieved to make a final decision when disagreement occurred between readers inside the group. Readers in each group were aware the patients were diagnosed with NSCLC, whereas blinded to the manifestations of the opposite modality and pathological findings. Intra- and extra-pulamonary anatomic structures studied including the size of tumor, involvement of adjacent organs or chest wall, and lung metastases were evaluated according to the 7^th^ Edition of the TMN Classification for Lung and Pleural Tumors. Visceral pleural invasion were identified as any contact between the tumor and pleura with associated pleural thickening or retraction, whereas the visualized extra-pleural fat plane was present. The contact between the mass and chest wall exceeding 3 cm or more, presence of obtuse angle between the mass and the chest wall, mass extending into the chest wall, loss of the extra-pleural fat plane, and rib destruction were the criteria in the assessment on chest wall invasion [7]. The following criteria was used in the identification of mediastinal invasion: extensive contact between the tumor and mediastinum, pleural and pericardial thickening, contact of 180° or greater between the tumor and mediastinal central vessels, and loss of the fat plane between the tumor and the mediastinal structures [8, 9]. The assessment of T staging then was compared with the diagnosis proven by pathology for each lesion.

### Statistical analysis

Statistical analyses were performed with SPSS 13.0 software. Accuracies of MRI and MDCT were calculated by using the standard definitions. Confidence intervals (CIs) were calculated for accuracy values on the basis of a 95 % confidence level. The differences of accuracy between MRI and MDCT were compared by using *McNemar* test. *p* value less than 0.05 was considered statistically significant.

## Results

Seventeen primary tumors were developed from the central areas (size range, 2.5-8.6 cm; mean size, 4.1 ± 1.6 cm) and 28 were originated from peripheral zone (size range, 1.3-8.3 cm; mean size, 3.4 ± 1.7 cm). The tumors were confirmed with pneumonectomy (*n* = 7), lobectomy (*n* = 32), and wedge-shaped resection (*n* = 6), respectively. These 45 patients were diagnosed with adenocarcinoma in 25, squamous cell carcinoma in 18, large cell neuroendocrine carcinoma in 2. T staging of the tumors were classified as T1 in 4, T2 in 28, T3 in 10, and T4 in 3, respectively. Surgical and pathological examinations of 45 patients revealed a diagnosis of pleural invasion in 22 patients, chest wall invasion in 4 patients, mediastinal invasion in 3 patients.Table 1 gives the clinical and pathological characteristics of 45 patients diagnosed with NSCLC.

**Table 1:** Clinical and Pathological Characteristics of 45 Patients diagnosed with NSCLC

Parameters	No. of patients
Total patients	45
Sex	
Male	30
Female	15
Age (y)	
>50	35
≤50	10
Diameter (cm)	
>3	37
≤3	8
Surgical procedure	
Wedge-shaped resection	6
Lobectomy	32
Pneumonectomy	7
Histological subtype	
Adenocarcinoma	25
Squamous cellcarcinoma carcinomacarcinoma	18
Large cell carcinoma	2
Site of invasion	
Visceral pleura	22
Chest wall	4
Mediastinum	3

Of the 45 primary tumors, T stages were correctly diagnosed in 38 (84.4 %) (The numbers in parenthesis stand for the accuracies, similarly hereinafter) patients (95 % CI: 64.2 %, 89.2 %) on MDCT, and in 37 (82.2 %) patients (95 % CI: 72.8 %, 94.6 %) on MRI. Table 2 provides the results of T stages in 45 patients with NSCLC using MDCT and MRI. Table 3 shows the comparison of efficacies of MDCT and MRI in T staging of 45 patients with NSCLC. Although no statistically significant difference was presented between the two modalities in the overall T staging of NSCLC when compared with that on the pathological findings (*p* = 0.564), MDCT was indicated more accurate in determination of NSCLC staged T1 and T2 (100 % vs 75 %, 96.4 % vs 82.1 %) when compared with that on MRI, whereas MRI was presented slightly superior in identification of NSCLC staged T3 and T4 (80 % vs 60 %, 100 % vs 33.3 %) when compared with that on MDCT.

**Table 2:** T staging of MDCT and MRI in 45 Patients with NSCLC

T staging	MDCT				MRI			
	T_1_	T_2_	T_3_	T_4_	T_1_	T_2_	T_3_	T_4_
T_1_ (*n* = 4)	4	0	0	0	3	1	0	0
T_2_ (*n* = 28)	1	27	0	0	2	23	3	0
T_3_ (*n* = 10)	0	3	6	1	0	2	8	0
T_4_ (*n* = 3)	0	1	1	1	0	0	0	3
Total (n = 45)	5	31	7	2	5	26	11	3

**Table 3:** Efficacies of MDCT and MRI for T Staging in 45 Patients with NSCLC

T staging	MDCT	MRI	*P value*
T_1_ (*n* = 4)	100 % (4/4)	75.0 % (3/4)	/
T_2_ (*n* = 28)	96.4 % (27/28)	82.1 % (23/28)	0.480
T_3_ (*n* = 10)	60 % (6/10)	80 % (8/10)	0.157
T_4_ (*n* = 3)	33.3 %(1/3)	100 % (3/3)	/
Total (n = 45)	84.4 % (38/45)	82.2 % (37/45)	0.564

**Fig. 1:**
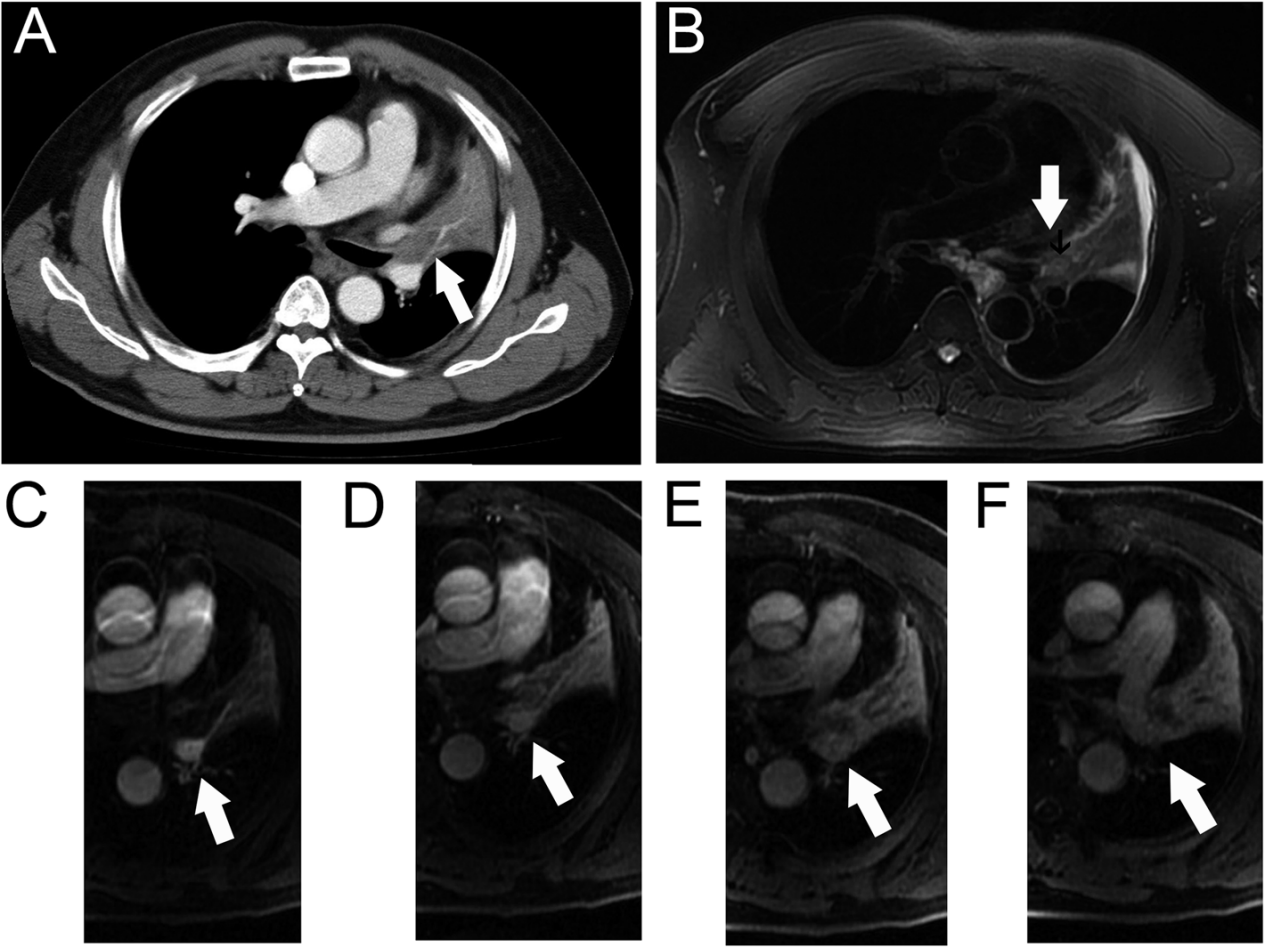
Images of 64-year-old man with diagnosed squamous cell carcinoma of the left upper lung. The extent of primary tumor in the left hilum (arrow) was not accurately distinguished from the secondary changes on axial MDCT (**a**). Tumor (arrow) was appeared as slightly hypointense compared the signal of secondary changes on axial T2-weighted MRI (**b**). Tumor (arrow) was showed as hypointense while that of secondary changes were hyperintense in early phase of dynamic contrast-enhanced MRI (**c** and **d**), in delay phase both of them were appeared as heterogeneously hyperintanse (**e** and **f**)

**Fig. 2:**
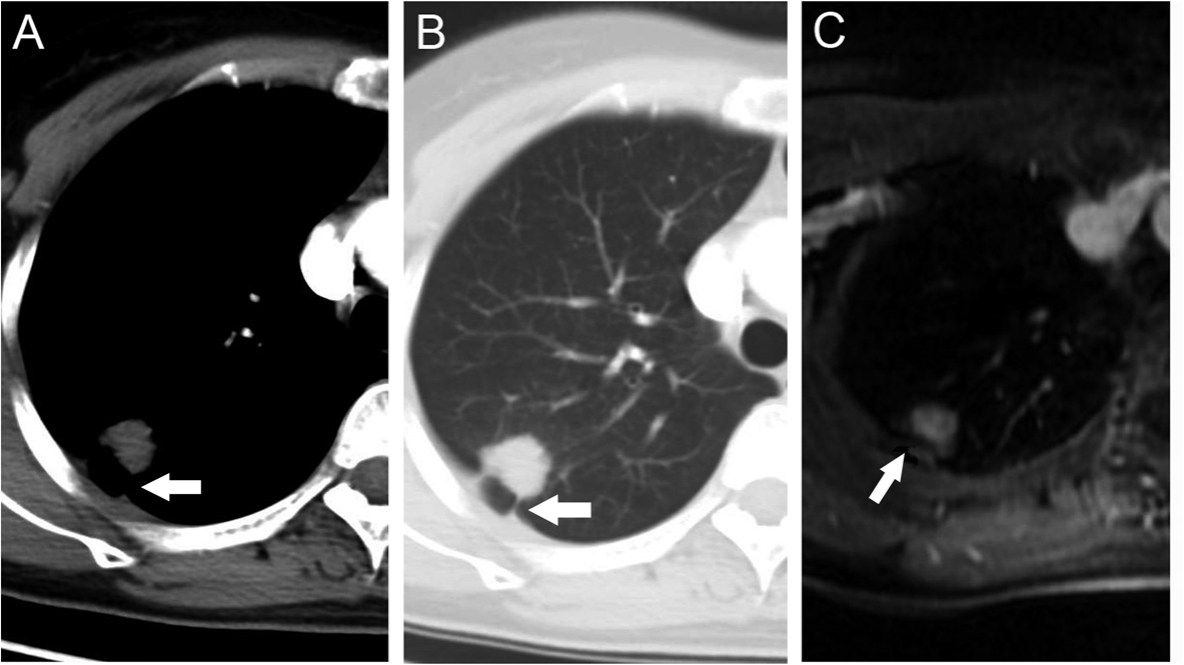
Images of 45-year-old woman with diagnosed adenocarcinoma of the right upper lung. Peripheral mass with associated pleural retraction (arrow) was identified on axial MDCT (**a** and **b**), suggestive of pleural invasion (tumor staged T2). Extrapleural fat plane (arrow) was identified on axial contrast-enhanced MRI (**c**). Pathology findings reported no evidence of visceral pleural involvement (tumor was staged T1)

**Fig. 3:**
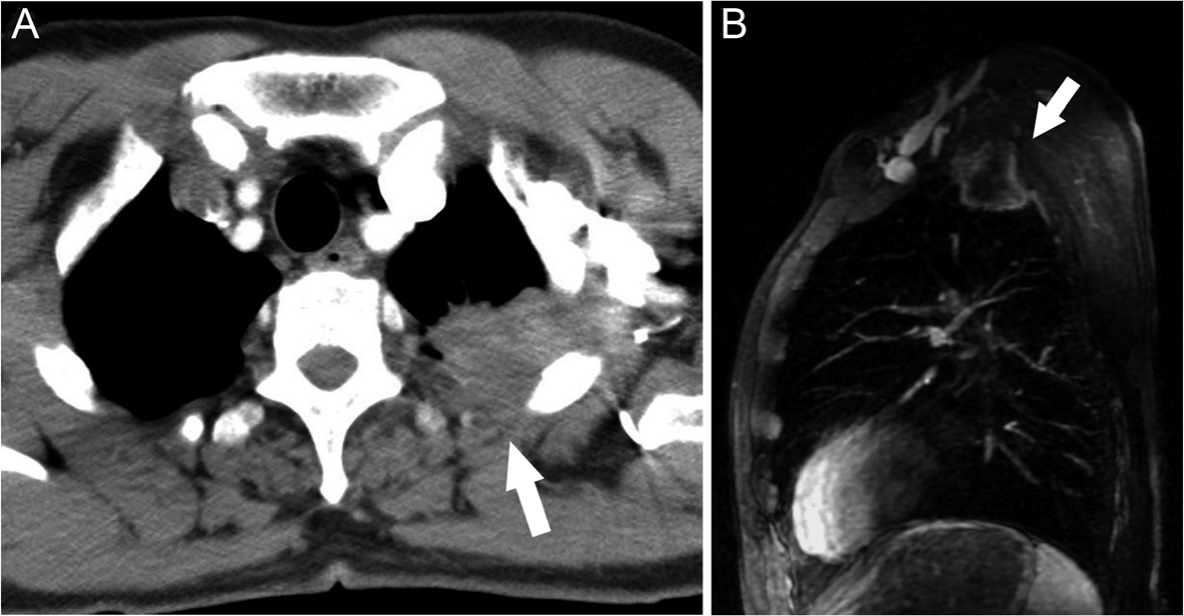
Images of 60-year-old man with adenocarcinoma of the left upper lung. Chest wall involvement (arrow) was identified on axial MDCT (**a**). Tumor was presented as heterogeneous enhancement and was invading into the extrapleural fat plan (arrow) on saggital image of contrast-enhanced MRI (**b**)

**Fig. 4:**
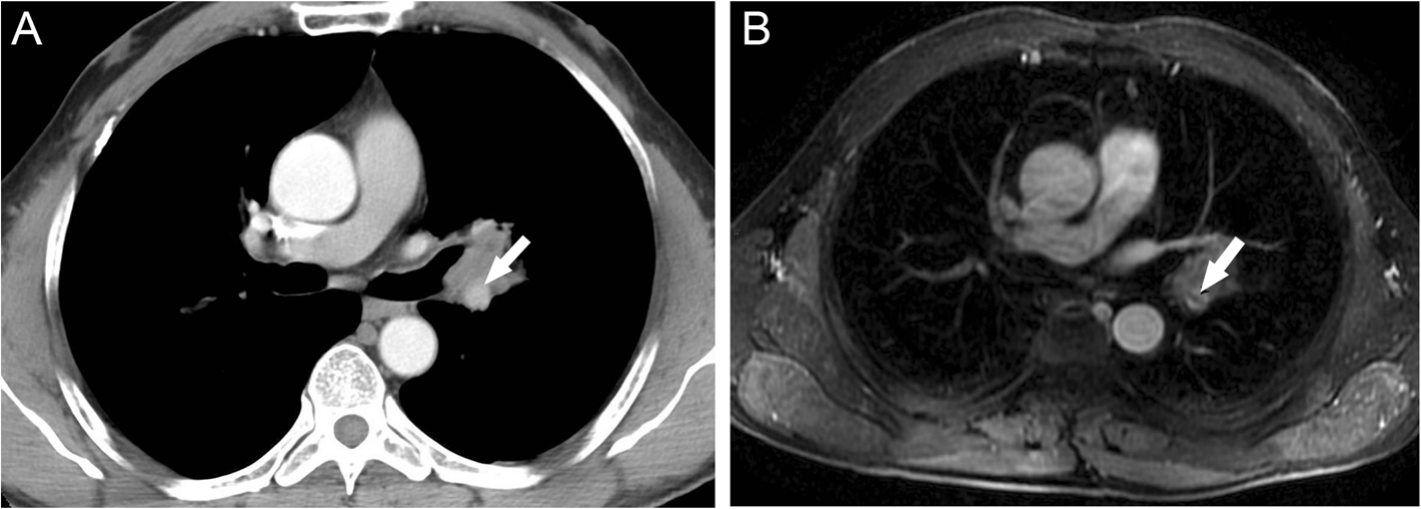
Images of 50-year-old woman with diagnosed adenocarcinoma of the left lung. Left pulmonary artery was visualized being encased with tumor tissue at less than 180° (arrow) which indicated no involvement of the vessel on transverse MDCT (**a**). Transverse contrast-enhanced MR image showed filling defect within the lumen of left pulmonary artery (arrow), caused the concern of tumor extending into the artery (**b**). The invasion was then confirmed with pathology (tumor was staged T4)

Secondary changes due to post-obstructive atelectasis or pneumonia was found in 26 patients, among them 16 (61.5 %) patients (95 % CI: 43.9 %, 81.1 %) were identified on MDCT, whereas 22 (84.6 %) patients (95 % CI: 70.7 %, 98.5 %) were determined on MRI (*p* = 0.014) (Fig. 1). Visceral pleural invasion of 22 cases was proved by surgical and pathological findings, 16 cases were seen on MRI while 12 cases were reported on MDCT (*p* = 0.003) (Fig. 2). Four cases were diagnosed with chest wall involvement on both modalities (Fig. 3). Three cases presenting mediastinal invasion were identified on MRI (Fig. 4); however, 2 of these 3 cases were misinterpreted using MDCT. Evidence of suspicious adrenal lesions was not noted in any case.

## Discussion

In this study, it was demonstrated that both MDCT and MRI provided acceptable overall accuracies in determination of T staging in the patients with NSCLC. Furthermore, MRI was presented slight superiority for the advanced-stage tumors (i.e., tumors staged T3 and T4), whereas MDCT was indicated more accurate for the limited-stage tumors (i.e., tumors staged T1 and T2) in the T staging of NSCLC.

Published research indicated that T staging in NSCLC was more correctly determinated on MRI than that on CT [10]. It was proved that 3.0 T MRI with the improved signal-to-noise ratio and higher contrast resolution in soft tissues combined with the application of phased-array cardiac coils increased the diagnostic efficiency in the assessment of T staging of NSCLC [11]. In our study, the overall accuracy for assessment of T staging in NSCLC on CT and MRI was 84.4 % (38/45) and 82.2 % (37/45), respectively, the difference between both modalities was not statistically significant, and was similar to the study reported by Yi et al, in which the primary tumors were correctly staged in 101 (82 %) patients on integrated PET/CT and in 106 (86 %) patients on whole body 3.0 T MRI [10].

In our study, involvement of mediastinal pleura (in that case tumor was staged with T3) of 2 cases were misrepresented on MDCT, which caused the underestimation of T staging in advanced tumor on MDCT, probably related to its relatively lower soft tissues contrast. Study has proved that MRI was more useful for distinguishing T3 and T4 tumors from early staged diseases owing to the superior contrast resolution [12]. One case of mediastinal pleura invasion proven by pathology was missed on MRI. It might be associated with the absence of cardiac-gated T1-weighted sequence applied in our study, as with this technique, MRI was considered being helpful in reducing the cardiac motion artifact that significantly influenced the evaluation of tumor involvement of areas of mediastinum [13]. In addition, 1 case with false positive of invasion of mediastinal pleura was occured on MRI, which might be due to the challenge to distinguish tumor invasion from benign inflammatory pleural adhesion on imaging modalities.

Contrast agents utilized in the developed MRI systems have shown some advantage on the staging of NSCLC. Previous research indicated that contrast-enhanced MRI was more useful in assessment of cardiovascular and mediastinal invasion than that on MDCT [14], the comparable sensitivity, specificity, and accuracy on MRI were reported up to 90 %, 87 %, and 88 %, respectively. Even though the advanced-stage cases included were limited, the advantages of enhanced MRI were also noted in our study: 3 cases associated with mediastinum invasion (presented as pulmonary artery involvement in this study) were accurately interpreted on MRI whereas only 1 of these cases was identified on MDCT. On the other hand, MRI was widely recognized to be more helpful in depicting tumor extent of the lung cancer arising from the central areas owing to the capability of MRI in distinguishing the tissues between the tumor and the associated atelectasis or pneumonia [15], as shown in the present study (*p* = 0.014). The signal representing primary tumor was noted significantly differed from that of secondary atelectasis and pneumonia (i.e., tumor manifested as hypointensity, whereas secondary changes presented as hyperintensity) on early phase of dynamic contrast-enhanced MRI. Four cases of central NSCLC with secondary changes confirmed by pathology were not revealed even on enhanced MRI, which might be related to the obstructive changes in the affected bronchus caused by limited-stage tumors were not sufficient to produce significant reduction of the air flow in the corresponding airways and lung tissues, thus were not visible on both modalities.

In this study, accuracy of diagnosis on determination of visceral pleural invasion (in that case tumor was staged with T2) assessed on MRI was statistically higher than that achieved on MDCT (*p* = 0.003). Although patient with chest wall invasion (in that case tumor was staged with T3) were used to the contraindication of surgical excision for lung cancer, studies have suggested that selected patients with chest wall infiltration who underwent surgical excision might be benefited with a better chance of survival by developed surgical technologies [16, 17]. Infiltration into the extra pleural fat, an associated pleural thickening, an obtuse angle, or more than 3 cm contact with the pleura are thought to be the sensitive but nonspecific findings in the diagnosis of chest wall invasion, the reliable manifestation suggestive of chest wall invasion is rib bone destruction. Previous studies have shown that sagittal and coronal MRI images with better tissue contrast resolution compared to that of CT were more effective in evaluating chest wall invasion [18, 19]. In this study, 4 cases of NSCLC presenting chest wall invasion were accurately diagnosed on either MRI or MDCT, for the latter, which might be contributed by the improved diagnostic capability of MDCT with multiplanar reconstruction capability [20]. For the case of superior sulcus tumor with involvement of surrounding structures (e.g., vessels), MRI has shown superiority of assessment of automatic relationship between the tumor and surrounding structure.

The distinction between tumors staged T3 and T4 on imaging has considered being clinically significant as the T3 lesions are potentially resectable while T4 lesions are not. In the present study, one tumor initially staged T4 on CT was diagnosed as T3 on MRI which proven by pathology. Furthermore, none of lesions staged T3 by pathology misdiagnosed as T4 on MRI. However, two of 3 cases staged T4 on MRI then proven by pathology were misdiagnosed on CT. For the patients with advanced-stage diseases, accurate interpretation on imaging features might improve the outcomes in the patients who were classified as the candidates of surgery, and reduce the morbidity and mortality rates in the ones who were treated with conservative approaches other than aggressive resection.

Selection bias is one of the major and common limitations in the researches applied the study design similar to that utilized in our study. Only three patients with primary tumor staged T4 were enrolled, as pathological evidence are only available for the patients who underwent operation on a potentially resectable stage of diseases. Only eight tumors with a measurement less than 3 cm in size were included in our study, as fewer patients referred to our institution that a national cancer center in our country presented early stage disease. Patients with certain positive indicators which should be concerned in the staging of lung cancer such as metastatic nodule were not included in the study. Such selection bias had certain impact on the evaluation of diagnostic accuracy in the present study; further study characterized by an enlarged sample size should be performed to confirm the validation.

## Conclusions

In conclusion, both MDCT and MRI provided acceptable accuracies in preoperative T staging of NSCLC. Furthermore, MRI was presented slight superiority for advanced-stage tumors (staged T3 and T4), whereas MDCT was indicated mild acceptance for limited-stage diseases (staged T1 and T2).
